# A pathway from chromosome transfer to engineering resulting in human and mouse artificial chromosomes for a variety of applications to bio-medical challenges

**DOI:** 10.1007/s10577-014-9459-z

**Published:** 2015-02-06

**Authors:** Mitsuo Oshimura, Narumi Uno, Yasuhiro Kazuki, Motonobu Katoh, Toshiaki Inoue

**Affiliations:** 1 Chromosome Engineering Research Center, Tottori University, 86 Nishi-cho, Yonago, Tottori 683-8503 Japan; 2 Department of Biomedical Science, Institute of Regenerative Medicine and Biofunction, Graduate School of Medical Science, Tottori University, 86 Nishi-cho, Yonago, Tottori 683-8503 Japan; 3 Division of Human Genome Science, School of Life Sciences, Faculty of Medicine, Tottori University, 86 Nishi-cho, Yonago, Tottori 683-8503 Japan

**Keywords:** Microcell-mediated chromosome transfer, Human artificial chromosome, Mouse artificial chromosome, Chromosome engineering, Humanized model mouse, Gene-/cell-therapy

## Abstract

Microcell-mediated chromosome transfer (MMCT) is a technique to transfer a chromosome from defined donor cells into recipient cells and to manipulate chromosomes as gene delivery vectors and open a new avenue in somatic cell genetics. However, it is difficult to uncover the function of a single specific gene via the transfer of an entire chromosome or fragment, because each chromosome or fragment contains a set of numerous genes. Thus, alternative tools are human artificial chromosome (HAC) and mouse artificial chromosome (MAC) vectors, which can carry a gene or genes of interest. HACs/MACs have been generated mainly by either a “top-down approach” (engineered creation) or a “bottom-up approach” (de novo creation). HACs/MACs with one or more acceptor sites exhibit several characteristics required by an ideal gene delivery vector, including stable episomal maintenance and the capacity to carry large genomic loci plus their regulatory elements, thus allowing the physiological regulation of the introduced gene in a manner similar to that of native chromosomes. The MMCT technique is also applied for manipulating HACs and MACs in donor cells and delivering them to recipient cells. This review describes the lessons learned and prospects identified from studies on the construction of HACs and MACs, and their ability to drive exogenous gene expression in cultured cells and transgenic animals via MMCT. New avenues for a variety of applications to bio-medical challenges are also proposed.

## Microcell-mediated chromosome transfer

### Dawn of MMCT

**Fig. 1:**
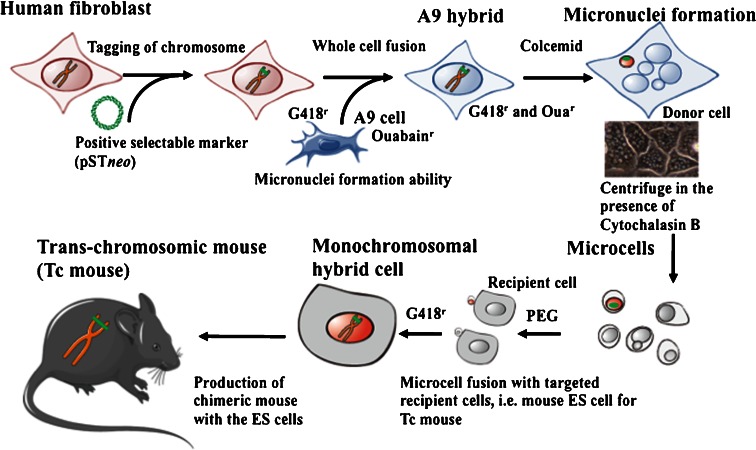
Microcell-mediated chromosome transfer (MMCT) for generation of monochromosomal hybrid cells and trans-chromosomic mice. Construction of mouse A9 hybrid cells carrying a single human chromosome by MMCT: the first step involves marking the human chromosome in the fibroblasts with a selection marker and fusing the fibroblasts with mouse A9 cells. The second step is the introduction of the marked human chromosome from the donor hybrid to the recipient A9 cells. The procedure can be divided into several parts, as follows: micronucleation of the donor hybrids by colcemid treatment, enucleation in the presence of cytochalasin B, purification of the microcells, fusion with the recipient A9 cells, drug selection of the microcell hybrids, identification of the transferred human chromosome by fluorescence in situ hybridization, and DNA analyses. This figure was produced using Servier Medical Art (http://www.servier.com)

Fournier and Ruddle performed for the first time microcell-mediated chromosome transfer (MMCT) (Fournier and Ruddle 1977). Several research groups have published seminal studies on the construction of mouse A9- or Chinese hamster ovary (CHO)-microcell hybrid libraries containing a single human chromosome tagged with a selectable genetic marker for MMCT (Fig. 1). A human chromosome tagged with a dominant selectable gene in the microcell-hybrids can be transferred to other cells. Therefore, the microcell hybrids provide valuable resources not only for mapping and cloning human genes but also for functional studies of specific genes and the production of animal models (Tomizuka et al. 1997; Shinohara et al. 2001; Meaburn et al. 2005; Devoy et al. 2011).

Briefly, donor cells (normally mouse A9 fibroblast cells and CHO) cells are induced to multinucleate their chromosomes (referred to as micronucleus formation). Micronuclei are then forced through the cell membrane to create microcells by centrifugation in the presence of cytochalasin B which disrupts the cytoskeleton (Ege and Ringertz 1974). These microcells can be fused to a recipient cell line in the presence of polyethylene glycol (PEG) which acts as a dehydrating agent and fuses plasma membranes. Thus, MMCT consists of two technologies, cell fusion and multinucleation. Since the basic procedure was established in 1970s, the essential part of the procedure has not changed.

The formation of multi-micronuclei is thought to be related to the condensed, scattered chromosomes that result from mitotic arrest caused by microtubule inhibitors. These scattered chromosomes reportedly serve as sites for the reassembly of nuclear membranes, forming micronuclei in which chromosomes decondense before returning to a pseudo-G1 phase (Crenshaw et al. 1981).

Cancerous or spontaneous micronuclei, which serve as an indicator of chromosomal instability, are small extra nuclei apart from primary nuclei in the same cell and are thought to be formed in an essentially similar manner to multi-micronuclei formed in A9 and CHO cells. However, multi-micronuclei and cancerous micronuclei differ in their number in a cell. The mechanism to create two different types of micronuclei in a cell-type-dependent manner remains unclear. Our observation suggests that A9 and CHO cells undergo repetitive hyperploidization in the presence of colcemid, and recurrent micronucleation occurs during transition from metaphase to pseudo-G1 to form smaller and more numerous micronuclei (Nakayama et al. 2015), whereas cancerous micronuclei formation is often associated with cell death. Thus, it appears that A9 and CHO cells have a system to escape from cell death provoked by mitotic error.

### A variety of applications of MMCT technique

MMCT technique has been applied to various types of studies since the 1970s. First, MMCT has contributed to mapping of genes whose defects resulted in clear cellular phenotype as shown by functional complementation studies (Meaburn et al. 2005; Doherty and Fisher 2003). This type of application includes gene mapping/isolation for tumor suppression (Oshimura and Barrett 1997), DNA repair (Matsuura et al. 1997, 1998; Horibata et al. 2004), metastasis, telomerase regulation, and genomic instability (Matsuura et al. 2006), mitochondrial disorders (Seyda et al. 2001), and lysosomal storage diseases (Kurimasa et al. 1993). Second, it has been applied to recapture and analysis of specific chromosome status in situations such as aneuploidy and Down syndrome (DS) and in epigenetics (Devoy et al. 2011; O’Doherty et al. 2005; Kazuki et al. 2014). Third, it has been applied to chromosome function study such as kinetochore assembly, telomere function, and high-order chromosome architecture (Wakai et al. 2014; Kouprina et al. 2014). Fourth—a new type of application of MMCT of mammalian artificial chromosome vectors—has recently been reported such as humanized animal transgenesis and cell and gene therapy, multi-reporter assay system, and high-yield protein production (Kazuki and Oshimura 2011; Oshimura et al. 2013). This fourth application will be further discussed later, while here we will now present representative applications of MMCT of the other three types.

#### Identification of tumor, metastasis, and telomerase suppressor genes

The studies using MMCT technique applied to human cancer cell lines have shown that tumor suppressor genes locate on Chr. 1, 3-13, 17-19, 22, and X (Oshimura and Barrett 1997; Doherty and Fisher 2003). The transfer of these chromosomes leads to growth suppression in vitro or decreased tumorigenicity in vivo. Since it is difficult to obtain and analyze the cells with growth suppression, revertants with spontaneous deletion on the transferred chromosome have often been used to identify the responsible region(s). Although the responsible genes on each chromosome have not yet been identified in many cases, recent advances in DNA sequencing technology would help the identification. Also, a combination of RNAi/genome-editing techniques (Joung and Sander 2013; Sander and Joung 2014) for knockdown/knockout of candidate gene(s) on the defined chromosome with MMCT technique would be useful. For example, the genes or regions on the transferred chromosome, whose knockdown or knockout in chromosome-transferred cells reverse the phenotypes of growth and tumorigenicity can be candidates for tumor suppressor genes/regions.

As well, metastasis-suppressor genes have been reported, which locate on Chr.1, 2, 6–8, 10–13, 16,17, and 20 (Doherty and Fisher 2003; Cheung et al. 2009; Ichikawa et al. 1994). Breast cancer metastasis-suppressor 1 (BRMS1), encoded at chromosome 11q13, has been identified as a breast carcinoma metastasis-suppressor gene (Seraj et al. 2000).

Telomerase, a ribonucleoprotein enzyme that maintains telomere length, is crucial for cellular immortalization and cancer progression. The loss of the activity sometimes leads to growth suppression in cancer cell lines. By essentially the same approach as identification of tumor suppressor regions, the identification of telomerase suppressor genes has been performed (Oshimura and Barrett 1997; Tanaka et al. 1998; Nishimoto et al. 2001; Kugoh et al. 2003; Abe et al. 2010). To date, telomerase suppressor genes have been found on almost all human chromosomes. Notably among the studies, Kugoh et al. has successfully identified PITX1—a member of the bicoid-related homeobox transcription factors—as a telomerase suppressor gene located on human chromosome 5 (Qi et al. 2011). The authors performed complementary DNA (cDNA) microarray analysis using parental telomerase-positive melanoma cells, telomerase-negative cell hybrids with a transferred human chromosome 5, and its revertant clones with reactivated telomerase. Thus, this study supports the notion that a combination of the latest biotechnology with the MMCT technique can lead to functional identification of genes as mentioned above.

#### Aneuploidy

A change in chromosome number, referred to as aneuploidy, is commonly observed in tumors. The observation that chromosomal aneuploidies arise in a tumor stage-specific manner suggests that they play a fundamental role in tumorigenesis. However, the relationship between aneuploidy and cancer remains unclear. For example, it is not known whether chromosomal aneuploidy affects chromosome-specific gene expression and whether it also affects gene expression on other chromosomes. MMCT methodology allows one to model specific chromosomal aneuploidies in cancer cells.

Three different chromosomes have been introduced into karyotypically diploid, colorectal cancer cells, and into immortalized normal breast epithelial cells (Upender et al. 2004). Their study showed that regardless of chromosome or cell type, chromosomal trisomies lead to a significant increase in the average transcriptional activity of the trisomic chromosome. In addition, this increase affects the expression of numerous genes on other chromosomes as well, suggesting that a complex pattern of transcriptional dysregulation exists in aneuploidy. Also, our group had established aneuploidy in mouse embryonic stem (ES) cells by transferring various human chromosomes or spontenous mouse chromosmal abnormalities and revealed a common cluster of down-regulated genes independent of the transferred human chromosome, of which eight known genes are related to cell proliferation, neurite outgrowth, and differentiation (Kai et al. 2009). This suggests that autosomal imbalance may commonly lead to dysregulation of apoptosis.

Recently, in vitro chromosome abnormality syndrome models with a genetic alteration were generated by combining chromosome transfer and genome-editing technologies (Kazuki et al. 2014). To gain insights into the underlying mechanisms of the progression to transient abnormal myelopoiesis (TAM) in DS patients, human pluripotent stem cells harboring Trisomy 21 (Ts21) and/or GATA-binding protein 1 shorter isoform (GATA1s) were generated via MMCT and zinc-finger nucleases (ZFN). The DS model cells generated by these two technologies are useful in assessing how GATA1s mutation is involved in the onset of TAM in patients with DS. Transfer of the modified chromosome or human artificial chromosome (HAC)/mouse artificial chromosome (MAC) with desired gene(s) via MMCT and disruption of a target gene(s) via genome editing will enable the identification of genes responsible for disease phenotypes.

#### Epigenetics

Epigenetics refers to heritable changes in gene expression that do not involve changes in DNA sequence. DNA methylation, histone modification, chromatin remodeling, transcription factors, and non-coding RNAs are currently considered to regulate epigenetic change (Bird 2007).

Genomic imprinting is the phenomenon of parent-of-origin gene expression. Appropriate expression of imprinted genes is important for normal development; their dysregulation is associated with numerous diseases such as Beckwith–Wiedemann syndrome, Angelman syndrome, and cancer. Our group has established a series of human monochromosomal hybrids housed in mouse A9 cells by using MMCT (Kugoh et al. 1999). Since the parental origin of the transferred chromosome is known, this library contributed in identifying a number of imprinted genes including LIT1, a long non-coding RNA gene involved in Beckwith–Wiedemann syndrome (Mitsuya et al. 1999; Meguro et al. 2001).

Chromosome 11 carrying LIT1 locus was transferred to homologous recombination-proficient chicken DT40 cells for targeted modification of the LIT1 genome, and then further transferred to CHO cells for the expression analysis of imprinted gene on the 11p15.5 region. This study successfully identified a putative imprinting control element playing an essential role in Beckwith–Wiedemann syndrome (Horike et al. 2000). The latest genome-editing technology described in the following section would allow direct genome modification in monochromosomal hybrids including A9 and CHO cells in much less time and with narrowing down of the region of interest without using DT40 cells. Also, monochromosomal hybrids still provide a good model to study chromatin organization on a centromeric region (Fukagawa et al. 2004).

### Towards a high efficiency of MMCT

Since the dawn of MMCT trials, established cell lines such as mouse ES cells have been preferably used as recipient cells. Efficiency of MMCT has been described by the ratio of drug-resistant colony number to recipient cells. Depending on the type of recipient cells, the efficiency is generally 10^−5^∼10^−6^ when using PEG for fusion of microcells and recipient cells. With the advent of stem cell studies, the target of MMCT has been extended to primary cells with finite life-span, somatic stem cells, and induced pluripotent stem (iPS) cells. Improvement of the efficiency has emerged as an issue to be resolved.

As mentioned in the previous section, MMCT comprises multiple steps, viz., (1) induction of micronuclei in donor cells, (2) isolation of microcells from micronucleated donor cells, (3) fusion of microcells with recipient cells, (4) integration of the transferred chromosome into host cell nucleus, and (5) selection of microcell hybrid cells by drug selection. Eventual efficiency is determined by the summation of contribution from each step. Of these steps, we will discuss microcell fusion and provide a perspective for effective isolation of microcells.

#### PEG fusion

PEG is most commonly used as a fusogen in microcell fusion and whole cell fusion by which monoclonal antibody-producing hybridomas have been routinely made (Yang and Shen 2006). Depending on the type of recipient cells, the microcell fusion efficiency where PEG is used is usually 10^−5^∼10^−6^. The mixture of microcells and recipient cells is transiently exposed to PEG, followed by dilution and removal by washing. Theoretically, one-to-one fusion between a pair of microcell and recipient cell produces a seed of microcell hybrid. But the PEG treatment is injurious to cell membrane, and overexposure to PEG reduces the viability of hybrid cells (Golestani et al. 2007). Furthermore, exposure of cells to PEG is difficult to precisely control in practice. As a common feature of established cell lines is infinite proliferation capacity, drug-resistant hybrids could easily arise, regardless of damage caused by PEG exposure. MMCT by PEG has been attempted with primary human fibroblasts or bone marrow-derived mesenchymal stem cells, but hybrids had been scarcely obtained (unpublished data). Substitution of fusogen from highly toxic PEG to less-toxic reagents might aid in generating microcell hybrids from primary cells.

#### Virus fusion

The first microcell cell fusion experiment was carried out by using Sendai virus as fusogen (Fournier and Ruddle 1977). But from difficulties in preparation of virus particle, virus fusion had been replaced with more convenient PEG fusion. Envelope-typed virus, which is coated by lipid bilayer membrane inherited from the infected host cells, makes use of an envelope protein(s) for the infection into next host cells. Sendai virus represents two distinct glycoproteins on the envelope for infection (Okada 1993). Hemagglutinin neuraminidase protein binds to sialic acid receptors on the host cell surface and degrades the receptor by sialidase activity. Fusion protein then associates with lipid molecules, such as cholesterol, embedded in the lipid bilayer membrane, and induces cell fusion. Utilization of inactivated virus particle for whole cell fusion had been reported since 2004 (Hiraoka et al. 2004), due to appearance of commercially available reagent. Application of the inactivated virus particle has also been reported for microcell fusion (Yamaguchi et al. 2006; Nawata et al. 2011; Lee et al. 2013). However, efficiency of MMCT was comparable but not superior to when PEG was used, even though immortalized cells were used as recipients. Unwanted secondary fusion between microcell hybrids and adjacent cells might hamper the survival of microcell hybrids.

#### Fusion by viral fusogenic proteins presented on the microcell surface

During the course of virus particle formation in the infected cells, viral fusogenic proteins are synthesized de novo, transported to the cell periphery, and presented on the cell surface, followed by extrusioin of cell membrane as virus envelope (Navaratnarajah et al. 2009). This well-controlled budding mechanism of envelope virus prompted us to make “a fusogenic microcell” which carries a chromosome to be transmitted and is coated with fusogenic envelope proteins. We chose fusogenic envelope proteins from measles virus (MV) with which accumulating data has been reported. MV has two envelope glycoproteins, hemagglutinin (H) and fusion (F) proteins, for infection into host cells. Virus particle specifically attaches to the surface of host cells by the interaction between H protein and its receptors on the host cell surface. Binding of the H protein to a receptor triggers the fusion of virus envelope with the host cell membrane by the mediation of F protein. To make “fusogenic microcells,” expression plasmids encoding H and F protein were transfected into CHO cells carrying a HAC vector. Microcells isolated from the CHO donor cells showed fusion ability to recipient human cells that express a receptor protein CD46, leading to successful transfer of the HAC (Katoh et al. 2010). It was noted that the MMCT efficiency depended on the expression level of CD46 in recipient cells. CD46 belongs to the family of complement activation regulators that prevent self-cell destruction (Dhiman et al. 2004). Overexpression of CD46 is frequently observed in cancer cells to overcome lysis by complement (Anderson et al. 2004). Indeed, in case of fibrosarcoma cell line HT1080 which has high surface density of CD46, the MMCT efficiency was 2 orders in magnitude higher than that of PEG fusion. However, in case of primary fibroblasts that have low surface density of CD46, the efficiency was comparable with that of PEG fusion. An issue with this method is the narrow range of recipient cells that can be used to obtain high efficiency fusion. The preceding studies that describe the usage of MV for oncolysis by infection, have proposed retargeting of MV by engineering the H protein, i.e., addition of single-chain antibody fragment against surface receptors other than CD46 (Nakamura et al. 2004; Nakamura et al. 2005). Addition of single-chain variable fragment (scFv) against transferrin receptor (TfR) improved fusion efficiency to primary fibroblasts (unpublished data). Although high affinity scFv to desired surface receptor is not always available with ease, retargeting of MV may be an alternative for PEG-sensitive cells.

### Isolation and storage of microcells

Microcell population prepared from donor cells is composed of a variety of subpopulations carrying (1) a chromosome (HAC or MAC) to be transferred, (2) chromosomes derived from host cells, or (3) no choromosomal DNA. Only the first subpopulation contributes to intended MMCT, but it is minor among all the components. In the current protocol, total mixed population is used for fusion with recipient cells, and intended microcell hybrids are chosen by selection culture with antibiotics by utilizing the drug-resistant gene tagged onto the HAC/MAC. Preferential fractionation of required microcell subpopulation from the total mixture, if possible, might aid in achieving more intentional fusion reaction and in needing a smaller number of recipient cells.

The conventional MMCT method is usually performed immediately after the purification of microcells. The timing of the isolation of microcells and the preparation of recipient cells is very important. A cryopreservation method to store microcells at −80 °C was performed and compared the efficiency of MMCT with conventionally (immediately) method. There was no significant difference between the two methods regarding chromosome transfer efficiency. Thus, cryopreservation of ready-to-use microcells is useful for the MMCT (Uno et al. 2013).

In order to isolate a single microcell-containing HAC/MAC, potential clues might be emerged from recent advancement in genome engineering technology, including zinc finger protein (ZFP), transactivator-like effector (TALE), and clustered regulatory interspaced short palindromic repeat (CRISPR)/Cas9 system (Urnov et al. 2010; Joung and Sander 2013; Sander and Joung 2014). Dynamics of a specific chromosome locus in living cells becomes detectable by tagging with DNA/RNA-binding protein fused with fluorescent proteins. Both long repetitive sequences such as telomeres or satellite centromeric DNA and short repetitive sequences such as within the intron of endogenous *MUC4* gene were visualized by the fusion of a fluorescent protein with TALE or CRISPR/Cas9 (Miyanari et al. 2013; Ma et al. 2013; Chen et al. 2013). In donor cells, centromere satellite of HAC/MAC is distinct from that of host chromosome; HAC/MAC-specific tagging at centromere may therefore be an attractive option. Microcells carrying HAC/MAC might be fractionated by FACS technology and efficiently transferred to desired cells or to a small number of cells, if they were specifically tagged with fluorescent fusion proteins utilizing genome engineering technology.

### Various types of HACs and MACs as episomal vectors

#### Transition of the cargo in MMCT from whole chromosome to HAC/MAC

In transferring a single chromosome or fragment, it is difficult to uncover the function of a specific gene because each chromosome or fragment contains a set of numerous genes. Thus, alternative tools are HAC and MAC vectors, which can carry a gene or genes of interest.

**Fig. 2:**
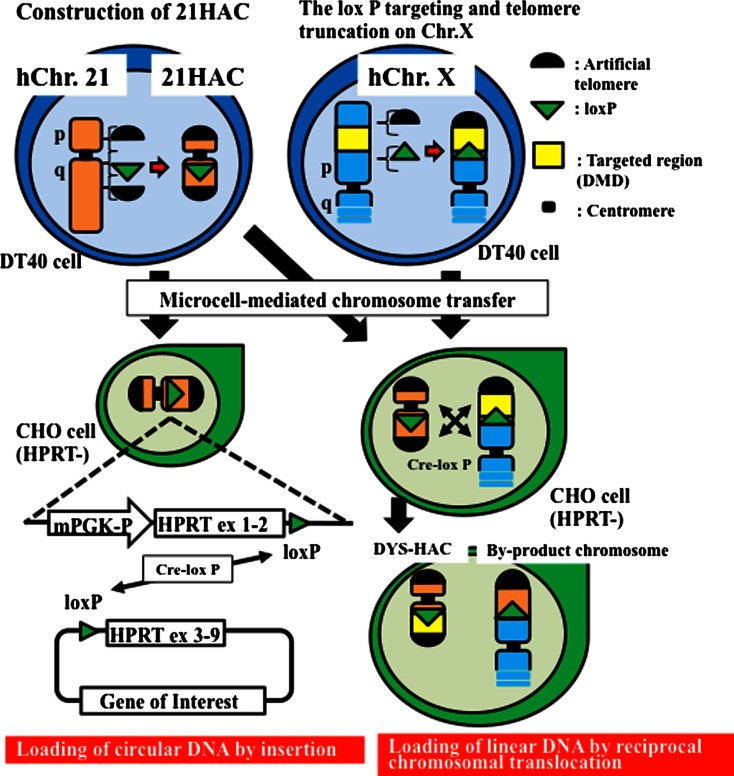
Two types of gene loading to HAC. (a) Construction of a human artificial chromosome (HAC) vector from human chromosome 21 using the top-down approach. The 21HAC is equipped with a *loxP* site for loading the gene of interest. A site-specific recombination event mediated by Cre recombinase is selected by reconstruction of the functional *HPRT* gene, which confers hypoxanthine-aminopterin-thymidine (HAT) resistance. (b) The gene of interest, isolated in a circular vector, is introduced into the HAC by site-specific insertion. (c) A megabase-size gene locus, which is above the capacity of circular cloning vectors, is introduced into the HAC by site-specific reciprocal chromosome translocation

Most, but not all, conventional vectors present problems associated with their limited cloning capacity, lack of copy number control, and insertional mutagenesis caused by integration into host chromosomes (Kouprina et al. 2014; Kazuki and Oshimura 2011). HACs and MACs are exogenous mini-chromosomes artificially created by either a top-down approach (engineered creation) or a bottom-up approach (de novo creation). In chromosomes engineered by a top-down approach, mini-chromosomes are derived from endogenous chromosomes following their natural fragmentation or telomere-directed chromosome breakage (Heller et al. 1996; Kazuki et al. 2011; Takiguchi et al. 2012). The HAC/MAC can then be transferred into other cell lines by MMCT. In de novo artificial chromosomes engineered by a bottom-up approach, exogenous chromosomes can be circular or linear, created de novo from cloned chromosomal components that possess a functional centromere, and can autonomously replicate and segregate. A summary of various chromosomal vectors and their acceptor site(s) and characteristics is provided in Table 1. The recent demonstration that chromosomal vectors can incorporate a gene or genes has increased their utility and potential application (Kouprina et al. 2014; Kazuki and Oshimura 2011; Oshimura et al. 2013) (Fig. 2).

**Table 1:** A list of HACs/MACs with various acceptor site(s) for gene delivery (modified from Kazuki et al. 2011)

Name of HACs	Construction method	Origin of centromere	Insertion sites (copy number of the insertion site)	Reference
Tet-O HAC	De novo	Human chromosome 17 alphoid	loxP (single/multiple), SIM system (loxP/attB/attP)	Iida et al. (2010)
25–4 vector	De novo	Human chromosome 21 alphoid	Mutant lox 71 (multiple)	Ikeno et al. (2009)
21ΔpqHAC, 21ΔqHAC	Engineered	Human chromosome 21	loxP (single)	Katoh et al. (2004)
21HAC1, 21HAC2, 21HAC3, 21HAC4	Engineered	Human chromosome 21	loxP (single)	Kazuki et al. (2010)
MI-HAC (21HAC1-modified HAC)	Engineered	Human chromosome 21	FRT, φC31attP, R4attP, TP901attP, Bxb1attP (single)	Yamaguchi et al. (2011)
Human mini-chromosome	Engineered	Human chromosome Y	attB (single)	Dafhnis-Calas et al. (2005)
CV (HCV/SAC)	Patient-derived accessory chromosome	Human chromosome 20	loxP (unknown copy number)	Voet et al. (2003)
MC	Patient-derived accessory chromosome	Human chromosome 9	loxP (5 copies)	Moralli et al. (2001)
SC20-HAC	Chromosome fragment	Human chromosome 14	loxP (single)	Kuroiwa et al. (2000)
14AΔqHAC, 14NΔqHAC, 14gNΔqHAC	Engineered	Human chromosome 14	loxP (single)	Kakeda et al. (2011)
SATAC	De novo (murine satellite DNA based)	Murine chromosome 7	loxP (multiple)	Stewart et al. (2002)
Platform ACE (SATAC)	De novo (murine satellite DNA based)	Murine chromosome	attP (multiple)	Lindenbaum et al. (2004)
MAC1, MAC2	Engineered	Murine chromosome 11	loxP (single)	Takiguchi et al. (2012)
MI-MAC (MAC2-modified MAC)	Engineered	Murine chromosome 11	FRT, φC31attP, R4attP, TP901attP, Bxb1attP (single)	Takiguchi et al. (2012)

#### HACs

HACs display a number of advantages over conventional vectors, e.g., they do not integrate into the host genome and the size of gene(s), which they can carry is not limited (Fig. 2). The de novo assembly of HACs using the bottom-up approach has been developed in human fibrosarcoma HT1080 cells (Harrington et al. 1997; Ikeno et al. 1998; Kouprina et al. 2003; Basu et al. 2005b). In most cases, de novo generated HACs range from 1 to 10 Mb in size. An issue of de novo HAC was the restriction of HAC formation to a single cell type HT1080. Recently, Masumoto and colleagues discovered that in HT1080 cells, the level of H3K9me3 on alphoid DNA is substantially lower than in other human cell lines (Ohzeki et al. 2012). In other types of human cells, heterochromatin enriched with H3K9me3 is assembled quickly on the transfected alphoid DNA array, thus preventing CENP-A retention and HAC formation. It has been shown that tethering of histone acetyltransferases to the input alphoid DNA arrays breaks this cell-type-specific barrier for de novo CENP-A assembly and allows assembly of other kinetochore proteins, thereby leading to HAC formation in a wide range of cell types. Other systems for the construction of HACs have been developed to rapidly create bacterial artificial chromosome (BAC)-based HACs using the red-recombination system from bacteriophage λ (Kotzamanis et al. 2005) or using a modified bacterial Tn5 transposon (Basu et al. 2005b). Utilization of invasive *Escherichia coli* systems may facilitate de novo HAC formation (Narayanan and Warburton 2003). A technique based on the HSV1 amplicon greatly improved de novo HAC formation protocols (i.e., much higher efficiency and applicability to many different cell lines other than HT1080) (Moralli et al. 2006). HACs have been generated in immortalized cell lines such as HT1080 but never in stem cells. Recently, de novo HACs were also generated in human ES cell lines (Mandegar et al. 2011). Thus, this technology is potentially suitable for a wide variety of applications.

Another HAC with a conditional centromere that includes the tetracycline operator (tet-O) sequence embedded in the alphoid DNA array has been generated (Nakano et al. 2008). This conditional centromere can be inactivated by expression of tet-repressor fusion proteins, resulting in loss of the tet-O HAC. Since the desired gene cannot be inserted into the tet-O HAC without an acceptor site such as loxP or FRT, the tet-O HAC vector was adapted for gene delivery and gene expression in human cells (Iida et al. 2010). Thus, a loxP cassette was inserted into the tet-O HAC by homologous recombination in chicken DT40 cells. The tet-O HAC with the loxP cassette was then transferred into CHO cells. It has been shown that the enhanced green fluorescent protein (EGFP) transgene was efficiently and accurately incorporated into the tet-O HAC vector. The EGFP transgene was stably expressed in human cells after transfer via MMCT, and the transgenes inserted into the tet-O HAC were subsequently eliminated from cells by loss of the HAC due to centromere inactivation. The tet-O HAC vector has significant advantages over other expression/cloning systems because it provides a mechanism to compare the phenotype of a mammalian cell with or without a functional copy of any cloned gene of interest.

Conversely, HACs engineered via the top-down approach can also be constructed by telomere-associated chromosome fragmentation techniques in the homologous recombination-proficient chicken cell line, DT40 (Buerstedde and Takeda 1991). Such an approach can generate mitotically stable, linear mini-chromosomes. Initially, mini-chromosomes ranging in size from 0.5 to 10 Mb have been produced from both the human X (Farr et al. 1992) and Y chromosomes (Brown et al. 1994). These mini-chromosomes retain a normal centromere and are mitotically stable in human cells with only minor rearrangements. A novel 21HAC vector in which known endogenous genes were absent was developed using the top-down approach from human chromosome 21 (Kazuki et al. 2011). This 21HAC was physically characterized using a transformation-associated recombination (TAR) cloning strategy followed by sequencing of TAR-BAC clones, confirming that no known endogenous genes remained in the 21HAC. Thus, the 21HAC vector contains four useful features: (1) it has a well-defined genetic architecture; (2) it is present episomally, independent of the host chromosomes; (3) it is mitotically stable in human cells in vitro; and (4) any desired gene can be cloned into the 21HAC using the Cre/loxP system in CHO cells or by a homologous recombination system in DT40 cells (Kazuki et al. 2011; Kouprina et al. 2014; Oshimura et al. 2013). Using the Cre/loxP system, gene cloning can be performed by insertion- or translocation-type cloning (Fig. 2). Circular vectors such as plasmids, P1-derived artificial chromsome (PAC) s and BACs containing desired genes can be inserted into the HAC vector by insertion-type cloning. Megabase-sized genes, which cannot be cloned into the circular vectors, can be cloned into the HAC vector using translocation-type cloning (Hoshiya et al. 2009). Furthermore, using the homologous recombination system, two different vectors, each containing a desired gene, were inserted sequentially into 21HAC1 by homologous recombination in DT40 cells. Two or more vectors containing desired genes can be inserted sequentially into the HAC. The genome-editing technology will also enable us to perform this recombination process easily without using DT40 cells. Therefore, any combination of genes, including full-length genomic DNA, can in theory be cloned into the HAC by a combination of these cloning systems and transferred into a desired recipient cell type using the HAC. Thus, these novel 21HAC vectors may be useful for gene and cell therapies as well as for animal transgenesis. The novel HAC vector may be generated using genome-editing technologies in human primary or iPS cells via top town approach for safe gene and cell therapy, without using intermediate host cells such as DT40 cells although the technologies for transfer of the HAC from normal human cells to desired patient cells without using A9 or CHO cells need to be developed.

#### MACs

Recently, a MAC vector was constructed from a natural mouse chromosome by means of the top-down approach (Takiguchi et al. 2012). In order to use the MAC as a functional gene vector, the faithful segregation of the MAC vector was investigated after its transfer to mouse embryonic stem (ES) cells and in trans-chromosomic (Tc) mice. Although human chromosome fragments (hCFs) and HACs with a large genomic region of interest could be autonomously maintained in Tc mice, their retention rate was variable in mouse ES cell lines and the tissues of Tc mice, possibly owing to their gradual loss during cell growth. On the other hand, MAC vectors are stably maintained in mouse ES cells and various tissues in Tc mice as well as in human cell lines (Takiguchi et al. 2012; Kazuki et al. 2013a). The MACs have acceptor sites into which a desired gene or genes can be inserted, similar to the HACs described above. Tc mice containing the MAC vector may be valuable tools for functional genome analyses in in vitro and in vivo models.

Furthermore, a satellite-DNA-based artificial chromosome (SATAC) was created via amplicon-dependent de novo chromosome formation induced by the integration of exogenous DNA sequences into centromeric DNA regions near the pericentric heterochromatic or acrocentric chromosome (Lindenbaum et al. 2004).

A mini-chromosome, ST1, was developed from the human Y chromosome, which is linear, has a molecular weight of approximately 4.5 Mb, and contains incidentally acquired mouse major and minor satellites as well as human DNA, including tandemly repeated alphoid DNA sequences (Shen et al. 1997). At the present time, the most suitable chromosome vectors reported in the literature remains uncertain, because comparative studies in the same condition (cell lines, mouse lines, culture method, etc.) has not been reported.

#### Technologies for multiple acceptor sites on chromosome vectors

One of the major problems with gene transfer into mammalian cells by standard methods of plasmid transfection or virus vector infection is poorly reproducible expression level of the transferred gene between different transformants because of chromosome position effect and copy-number variation. Targeted integration of DNA into the acceptor site on the chromosomal vectors promises a simple solution to the problems from random integration of the transferred gene.

Chromosomal vector is maintained in mammalian host cells. Prototype chromosomal vector utilizes the Cre/loxP system for site-specific insertion of circular donor vector into the cloning site on the chromosomal vector (Fig. 2). Donor vector carries a gene of interest, loxP cassette, and a part of a drug-resistant marker gene. Chromosomal vector carries acceptor loxP cassette and the other part of the drug-resistant marker gene. After co-transfection of the donor vector and the Cre expression plasmid into the host cells, site-specific insertion is correctly selected by reconstruction of the drug-resistant marker gene. In addition to dominant selectable markers like neo (Fukushige and Sauer 1992), hypoxanthine phosphoribosyl transferase (HPRT) mini-gene cassette (Ramírez-Solis et al. 1995) is useful in HPRT-deficient host cells, which are easily isolated as 6-thioguanine (6-TG) resistant mutants. One application of chromosomal vector is simultaneous transfer of multiple genes into target cells. Since the prototype chromosomal vector possesses a single-acceptor loxP site, multiple genes should be unified in a donor vector such as BAC or PAC by conventional in vitro recombinant DNA technique. Processing of BAC or PAC for unifying multiple genes is, however, laborious because of their low copy number in host *E. coli* and of their large size beyond the fractionation range in gel electrophoresis.

An alternative to unifying multiple genes in a single donor vector is increase of acceptor site on chromosomal vector by utilizing other integrase systems capable of site-specific insertion of donor vector. Application of several integrase systems derived from different microorganisms had been reported in mammalian cells (Fogg et al. 2014). Yamaguchi et al. made a HAC vector carrying five acceptor sites for utilizing FLP, φC31, R4, TP901-1, and Bxb1 integrases (Yamaguchi et al. 2011), which was designated as MI-HAC. In the MI-HAC, a pair of promoters for a selection marker gene and acceptor site for an integrase was tandemly placed, while in a donor vector, a gene of interest was placed along with promoter-less drug-resistant gene. Theoretically, up to five different genes of interest could be loaded onto the MI-HAC by reconstruction of the selection marker gene by site-specific integration of the donor vector.

In the case of a bottom-up HAC (25-4 vector), since multiple lox71 sites were integrated into the HAC, a lox66 sequence in the donor vector containing desired gene is useful for the sequential insertion of multiple desired genes into the HAC (Hasegawa et al. 2014). A drawback of this system is that the stability and germline transmission efficiency of the HAC was decreased after second gene insertion potentially due to the structural changes of the HAC.

In case of top-down HAC (49B(A)A9 mini-chromosome), iterative site-specific integration (ISSI) system on the 49B(A)A9 mini-chromosome derived from human Y chromosome were developed (Dafhnis-Calas et al. 2005). ISSI combined the activities of φC31 integrase and Cre recombinase to enable the iterative and serial integration of transgenic DNA sequences.

However, during the chromosome engineering processes for the construction of the chromosomal vector itself, several selection marker genes had been already used, and a limited number of selection markers are available for further integration of the donor vector into the chromosomal vector. To overcome the problem of this scarcity in selection markers, of relevance to loading of multiple genes to mammalian artificial chromosome, another example has been reported (Tóth et al. 2014). This approach is based on a protocol by which an artificial chromosome was made utilizing incidental chromosome rearrangement associated with the transfection of satellite DNA into murine cells (deJong et al. 1999). The artificial chromosome vector utilizes modified R4 integrase derived from lambda phage for target integration of donor vectors into the acceptor site on the artificial chromosome. By placing between two loxP sites in the donor vector, the selection marker gene can be excised by Cre enzyme expression after loading of a gene of interest, which allows sequential loading of different genes in other acceptor sites on the artificial chromosome vector. A drawback of this system is that the number of acceptor sites and loaded gene is not predictable.

To overcome the problem of this complicated gene-loading protocol on the chromosomal vectors, a simple method for the simultaneous or sequential integration of multiple gene-loading vectors into a HAC vector, designated as the simultaneous or sequential integration of multiple gene-loading vectors (SIM) system, was reported (Suzuki et al. 2014). In the SIM system, simultaneous integration is attained by stepwise nested insertion of gene-loading vector by different integrases. Sequential integration is attained by the shedding of formerly reconstructed marker gene, caused by targeted insertion of an ensuing donor vector. These are achieved by elaborate placement of target sequence for integration and smart utilization of splicing acceptor and donor cassettes to splice out the acceptor site for the next reaction embedded between the front and rear half of the selection marker genes. A prominent feature of this system is that multiple gene-loading vectors can be integrated by the cycling use of only two selection marker genes at most. Thus, the SIM system on HAC/MAC vectors is very useful and expected to expand the applicability of HAC/MAC vectors for multiple gene expression study, because SIM system can be applied to any HAC/MAC with a 5′HPRT-type cassette.

## Expression of genes in HAC/MAC

### Functional analyses

The functions of novel genes have been deduced mainly in experiments using viral transfection or integrative transfection of BAC or yeast artificial chromosome (YAC) vectors, where often the gene cannot be expressed at a physiological level because the gene copy number is not regulated and a BAC/YAC transgene randomly integrates into the host genome. The HACs and MACs described above provide a way to overcome these problems (Oshimura et al. 2013). Genes that have been loaded in various types of HACs/MACs are listed in Table 2. The alphoid^tetO^-HAC, which possesses a conditional centromere, provides a particularly effective way to control the phenotypic changes attributed to the expression of HAC-encoded genes. A battery of functional tests was performed to demonstrate the expression of the *NBS1* and *VHL* genes from the HAC at physiological levels, which showed that phenotypes arising from stable gene expression can be reversed when cells are “cured” of the HAC by inactivating its kinetochore in proliferating cell populations (Kouprina et al. 2014). Thus, this type of HAC should be suitable for studies of gene function. Exploiting the potential of HACs for further gene transfer and expression studies is the first step for subsequent proof-of-concept studies (Kim et al. 2011). Most recently, the HAC could be used for functional study of BRCA-1 tumor suppressor gene (Kononenko et al. 2014). Furthermore, two different HAC, a stable 21HAC and a removable tet-O HAC provides a unique bi-HAC vector system for transient gene expression (Iida et al. 2014).

**Table 2:** A list of HAC/MAC and their applications (modified from Kazuki et al. 2011)

Utilized HAC/MAC		Loaded genes	Aims	References
De novo HAC		Human GCH1	Expression of BAC-derived gene in vitro	Ikeno et al. (2002) and Suzuki et al. (2006)
		Human β-globin	Physiological expression of YAC-derived gene in vitro and in vivo	Suzuki et al. (2006, 2010)
		EGFP-derived from several promoters	Promoter-dependent gene expression	Ikeno et al. (2009)
		STAT3	Correlation of DNA methylation and gene expression	Ikeno et al. (2009)
		β-actin-SVLT	Treatment of non-albumin rats by immortalized hepatocytes	Ito et al. (2008, 2009)
		Human HGH/PDK1/β-globin	Expression of BAC-derived gene in vitro	Basu et al. (2005a)
		Human HPRT	Functional complementation of genetic deficiency	Mejia et al. (2001) and Moralli et al. (2006)
		Human HPRT	Functional complementation of genetic deficiency	Grimes et al. (2001) and Kotzamanis et al. (2005)
		Human factor IX	Expression of PAC-derived FIX gene	Breman et al. (2008)
		Human CFTR	Construction of HAC with entire CFTR gene	Laner et al. (2005) and Rocchi et al. (2010)
		Human BRCA-1	Funtional analisys of full-length of exogenous BRCA-1	Kononenko et al. (2014)
De novo HAC with loxP site(s)	25-4	HLA-DR loci (DRA and DRB1)	Multiple BACs insertions on the de novo HAC and generation of Tc mice	Hasegawa et al. (2014)
	21HAC2/tet-O HAC	Removable CAG-DsRed and CAG-EGFP	Collaboration de novo HAC with engineered HAC	Iida et al. (2014)
	Tet-O HAC	CAG-EGFP, tdTomato, and venus	Simultaneous or sequential integration of multiple genes	Suzuki et al. (2014)
CV (HCV/SAC)		Human CSN2	PAC genome insertion system	Voet et al. (2003)
MC1		CMV-human IL2	Cloning and expression of desired gene	Guiducci et al. (1999)
		Human CFTR	Functional expression of CFTR gene	Auriche et al. (2002)
SC20-HAC		Human IgH and Igk/Igλ	Production of humanized antibody	Kuroiwa et al. (2000, 2002, 2009)
		Human CYP3A cluster	Prediction of human drug metabolism and toxicity	Kazuki et al. (2013a)
21HAC	21ΔqHAC	Ubc-hTERT-IRES-GFP	Life-span extension of normal fibroblast	Shitara et al. (2008)
		PGK-ScFv-gp130-IRES-EGFP	Antigen-mediated growth control	Yamada et al. (2006) and Kawahara et al. (2007)
		TR-DNA-PKCs	Tetracycline-mediated inducible gene expression system	Otsuki et al. (2005)
	21ΔpqHAC	Mouse CD40L	BAC-PAC-mediated gene expression system for gene therapy	Yamada et al. (2008)
		CMV-EGFP	Expression of monitor gene/ transfer of HAC to human blood cells	Katoh et al. (2004) and Yamada et al. (2006)
		Human HPRT	TAR cloning-mediated or ready-made PAC-mediated gene insertion	Ayabe et al. (2005) and Kazuki et al. (2008)
		HSP70-insulin	Heat-regulated gene expression system	Suda et al. (2006)
		Human TP53	Genetic correction in mGS cells	Kazuki et al. (2008)
		OPN-EGFP	Lineage-specific gene expression	Ren et al. (2005)
		CMV-human EPO	Therapeutic protein expression in normal fibroblast	Kakeda et al. (2005)
		OC-GFP	Evaluation system for bioactive substances	Takahashi et al. (2010)
		OC-Luciferase	Evaluation system for bioactive substances	Narai et al. (2015)
	21HAC1	MC1-HSV-TK	Suicide gene- and MSC-mediated treatment of glioma	Kinoshita et al. (2010)
		CAG-EGFP	Expression of monitor gene	Kazuki et al. (2011)
	21HAC2	CMV-DsRed	Expression of second monitor gene	Kazuki et al. (2011)
		Human dystrophin	Gene therapy of DMD using 2.4 Mb dystrophin-HAC	Hoshiya et al. (2009), Kazuki et al. (2010), and Tedesco et al. (2011, 2012)
		Yamanaka factors and p53shRNA	Generation of iPS cells	Hiratsuka et al. (2011)
		CAG-human FVIII (1–16 copies)	Copy number-dependent gene expression system	Kurosaki et al. (2011)
		PF4-FVIII	Tissue-specific gene expression of FVIII to avoid immunorejection	Yakura et al. (2013)
	MI-HAC	CMV-EGFP	Gene insertion on multiple integration site(s)	Yamaguchi et al. (2011)
14HAC	14AΔqHAC	CMV-EPO, UBC-EPO, UL15-UBC-EPO	Gene expression analysis of 14HACs with various promoter	Kakeda et al. (2011)
	14NΔqHAC	UL4-UbC-Sox2-Klf4-c-Myc-Oct4	Generation of iPS cells	
	14gNΔqHAC	and ColEEP-Oct4		
SATAC	Platform GFP-ACE	CAG-human EPO	Production of pharmaceutical protein	Lindenbaum et al. (2004)
	Platform ACE	CAG-Human IgG1 antibody	Production of pharmaceutical protein	Kennard et al. (2009a, 2009b)
		CAG-murine/humanGALC	Model experiment for treatment of Krabbe’s disease	Katona et al. (2008)
		CAG-Oct4, CAG-Sox2, and CAG-Klf4	High level of protein production	Toth et al. (2014)
		CAG-hrGFP	Expression of monitor gene/ transfer of SATAC to human blood cells	Lindenbaum et al. (2004) and Vanderbyl et al. (2005)
	Derived from H1D3 and mM2C1	SV40-LacZ	Stability test of SATAC in different cell lines	Telenius et al.(1999) and de Jong G et al. (2001)
	Derived from A9	CMV-GFP	Flow-sorted chromosome transfer	Vanderbyl et al. (2001)
	D11-C4 ACE	CMV-RFP	Expression of monitor gene/ transfer of SATAC to hMSC	Stewart et al. (2002) and Vanderbyl et al. (2004)
MAC	MAC1	CAG-EGFP	Stability test of MAC in mice and human cells	Kazuki et al. (2013a)
	MAC2	CAG-EGFP	Gene insertion on loxP site	Takiguchi et al. (2012)
	MI-MAC	CAG-EGFP	Gene insertion on multiple integration site(s)	Takiguchi et al. (2012)

### Relationship between gene copy number and gene expression in HACs

A HAC vector, FVIII-HAC, carrying the human factor VIII (FVIII) cDNA, was constructed and inserted into CHO cells (Kurosaki et al. 2011). One or more copies of the FVIII gene on the HAC were expressed in a copy number-dependent manner in the CHO cells. The HAC with 16 copies of FVIII, FVIII (16)-HAC, was transferred from CHO hybrids into a human immortalized mesenchymal stem cell (hiMSC) line by MMCT. The expression levels of HAC-derived FVIII transgene products were compared with transfected FVIII plasmids. The results showed that the expression levels of the former were consistent with those of the original clones, even after 50 population doublings (PDLs), whereas the latter showed a remarkable decrease in expression despite a consistent DNA content. These findings showed that the gene on the HAC was resistant to gene silencing. As an example of the application for protein expression, SATAC was effectively used to rapidly generate stable CHO cell lines expressing high levels of monoclonal antibody (Kennard et al. 2009a; Kennard et al. 2009b). Thus, the HAC/MAC/SATAC-mediated therapeutic gene expression system may be a powerful tool for stable expression of transgenes and possibly for industrial production of gene products.

### Tissue-specific expression

The feasibility of lineage-specific transgene expression by the HAC vector was assessed in an in vitro differentiation system with an MSC cell line, hiMSC, which has the potential for osteogenic, chondrogenic, and adipogenic differentiation (Ren et al. 2005; Suda et al. 2006). An *EGFP* gene driven by a promoter for the osteogenic lineage-specific osteopontin (OPN) gene was inserted into the 21HAC and then transferred into hiMSC. The EGFP was specifically expressed in the hiMSCs that differentiated into osteocytes in coordination with the transcription of the endogenous OPN gene but was not expressed after adipogenic differentiation induction or in non-inducing culture conditions, indicating that the use of HAC vectors is suitable for regulated expression of transgenes not only in stem-cell-mediated gene therapy, but also in promoter analyses. Another application of tissue specific promoter is that erythropoietin (EPO) gene expression driven by the 5′ untranslated region of the human ubiquitin C gene on the newly developed chromosome 14-derived HAC greatly increased (over 1000-fold) the EPO production in hPFs (Kakeda et al. 2011). These tissue-specific expression patterns using HACs with their own promoters were also confirmed in Tc mice, including mice producing a fully humanized antibody (Tomizuka et al. 1997) and mice with the human cytochrome P450 enzyme (Kazuki et al. 2013b).

### Tc mice and humanized model mice

**Fig. 3:**
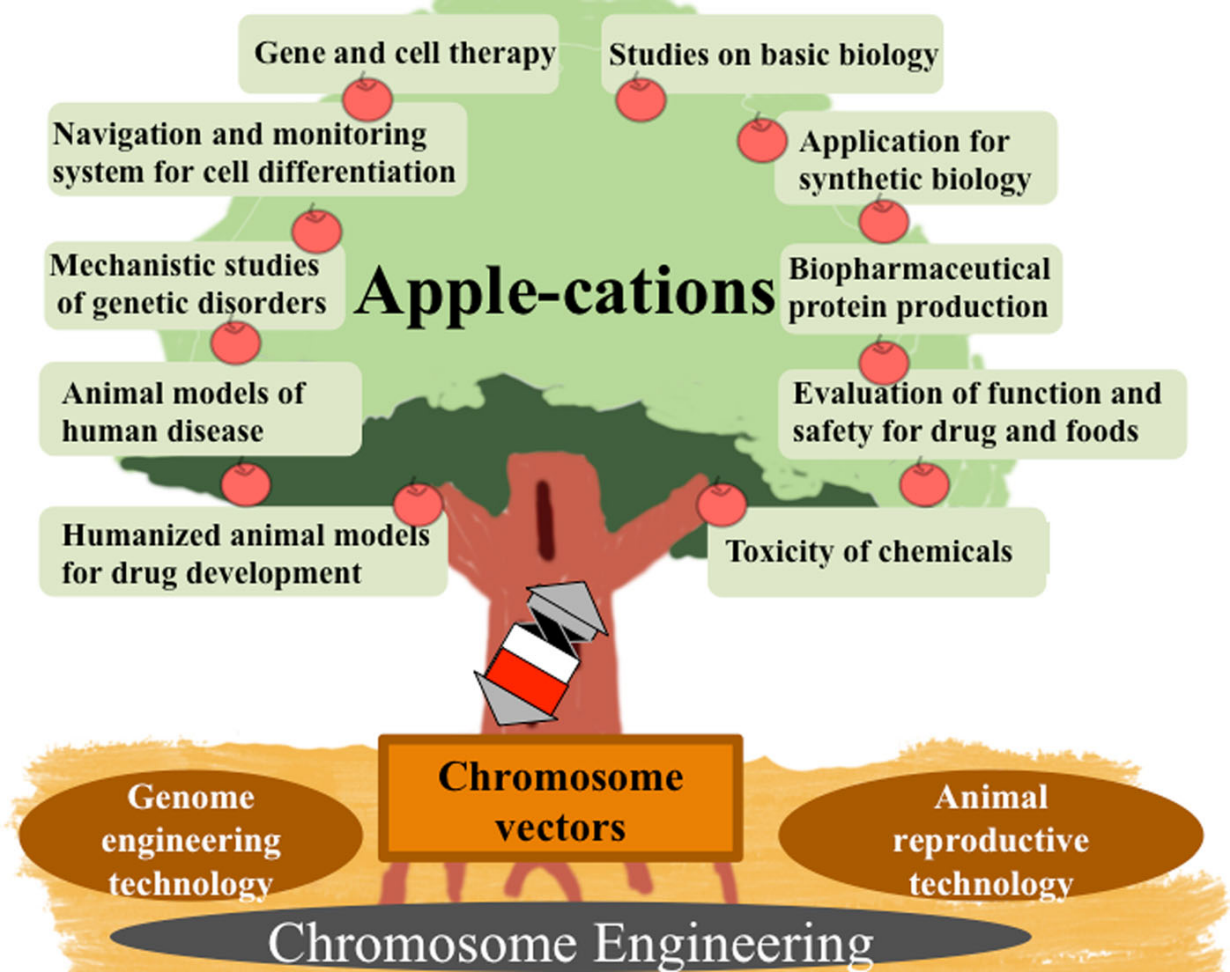
Fruits from applications of chromosome vectors in the bio-medical field

An increasing number of laboratories around the world employ the mouse as a model of human diseases (Devoy et al. 2011). Therefore, production of mice carrying human genes to model specific diseases or humanized functions is a potential future application of HACs/MACs (Fig. 3). One pre-requisite for these studies was the demonstration that HACs/MACs are mitotically stable not only in human cells but also in rodent cells.

Tomizuka and colleagues were the first to demonstrate the introduction of a hCF into mouse ES cells as well as in mice (Tomizuka et al. 1997). Specifically, they developed a chromosome fragment containing some 10-Mb-sized regions of chromosomes 2 and 14 carrying the *Igk* and *IgH* genes (Tomizuka et al. 2000). This hCF was stably maintained in mouse ES cells and subsequently in mice. Functional expression was obtained from the genes of the hCFs as well as the human antibody genes in mice. A similar technology was applied to cows to produce human immunoglobulin and antigen-specific human polyclonal antibodies from hyperimmunized cattle (Kuroiwa et al. 2009).

The other application of chromosome transfer technology is to generate Down syndrome model mice. Two groups have successfully generated Tc Down syndrome model mice (Shinohara et al. 2001; O′Doherty et al. 2005). These mice contain an extra human Chr. 21 and show cardiac abnormalities and behavioral impairment similar to patients with Down syndrome.

Another example is a humanized mouse with a human CYP3A cluster (Kazuki et al. 2013b). Human CYP3A is the most abundant P450 isozyme present in the human liver and small intestine, which metabolizes around 50 % of the medical drugs on the market. The human CYP3A subfamily comprises four members (CYP3A4, CYP3A5, CYP3A7, CYP3A43) encoded by a region on human chromosome 7. The introduction of a HAC containing the entire human genomic CYP3A locus recapitulates tissue- and stage-specific expression of human CYP3A genes and xenobiotic metabolism in mice. Thus, this system can be also used for generating Tc mice carrying a wide variety of other human metabolic genes. Since allelic expression imbalance of the human CYP3A4 gene was reported (Hirota et al. 2004), the desired two alleles converted from a CYP3A4 by the genome-editing technologies such as the CRISPR/Cas9 system can be transferred to a mouse to confirm the phenomenon observed in humans. Thus, the combination of HAC/MAC containing desired large genomic cluster and the genome editing will facilitate the generation of humanized animals.

The creation of transgenic mice using de novo constructed HACs carrying human beta-globin (Suzuki et al. 2006), *GCH1* (Suzuki et al. 2006), *CSN2* (Voet et al. 2003), and HLA (Hasegawa et al. 2014) genes also succeeded. These studies demonstrated that the HACs have been transmitted through the mouse germline, thereby providing evidence of the meiotic stability of the HACs in vivo. Thus, the proven availability of HAC vectors to carry certain genes in animals provides an opportunity to develop specific human disease models, and also to commercially produce therapeutic products. HAC/MAC-based transgenesis can be used to identify genes responsible for recessive phenotypes by complementation or expression of dominant phenotypes. This approach is also applicable to the study of the complex genomic network in a near endogenous context.

### Genes in HACs/MACs and medical applications

Gene therapy has been envisioned to provide a direct and permanent correction of genetic defects. To achieve the desired effects, therapeutic genes need to be carried by safe and effective vectors that can deliver foreign genes to specific cells and thereafter sustain their expression in a physiologically regulated fashion. Gene delivery vectors with the following properties may further add to the applications for gene and cell therapies: (1) high transfection efficiency; (2) long-term stable maintenance in host cells without integration into the host genome; (3) appropriate levels of spatial and temporal expression of therapeutic genes in specifically desired cells; (4) no risk of cellular transformation or stimulation of the host’s immune system; and (5) a system to safeguard against tumor formation. Although a number of different approaches have been attempted to achieve efficient gene transfer and long-term gene expression, this challenging task remains unfulfilled because all current methods have certain limitations, including transient expression, consequent toxicity, undesired immunological response, integration of target genes into the host cell genome, and transcriptional silencing. An alternative solution to these problems could be the use of HAC vectors. For example, the advantages of HAC vectors have been demonstrated for reprogramming mouse embryonic fibroblasts (MEFs) into iPS cells (Hiratsuka et al. 2011). A HAC carrying four reprogramming factors with a p53-knockdown cassette (iHAC) efficiently reprogrammed MEFs. Global gene expression patterns showed that the iHAC generated relatively uniform iPS cells. Under non-selecting conditions, iHAC-free iPS cells were isolated as cells that spontaneously lost iHAC2. Analyses of pluripotent markers, teratomas, and chimeras confirmed that these iHAC-free iPS cells were pluripotent. Moreover, iHAC-free iPS cells with a re-introduced HAC encoding *Herpes Simplex virus* thymidine kinase were eliminated by ganciclovir exposure, indicating that the HAC safeguard system functioned in iPS cells. Thus, the HAC vector could generate uniform, integration-free iPS cells with a built-in safeguard system (Uno et al. 2014).

Another example is HAC utility for gene therapy for Duchenne muscular dystrophy (DMD). *DMD* gene was newly loaded on 21HAC2 (Kazuki et al. 2010). DMD is caused by dysfunction of the dystrophin gene (Koenig et al. 1988). Since some DMD patients show a large deletion in the dystrophin gene, these defects cannot be corrected by exon-skipping approaches (Odom et al. 2007; Tedesco and Cossu 2012). Although several vectors have been developed for DMD gene therapy, no episomal vectors containing the entire dystrophin genomic region have been reported owing to the extremely large size of this region (2.4 Mb) (Koenig et al. 1987). Thus, a 21HAC vector containing the entire dystrophin genomic region (DYS-HAC) has been developed for potential application in DMD gene therapy (Hoshiya et al. 2009). The complete correction of a genetic deficiency was shown in iPS cells derived from DMD model (mdx) mice and a human DMD patient using the DYS-HAC. In addition, the DYS-HAC isoforms were verified in cardiomyocytes differentiated from iPS cells, which are derived from DMD patients (Zatti et al. 2014). More details are described by Tedesco in this special issue (Kazuki et al. 2010; Uno et al. 2014; Tedesco and Cossu 2012; Tedesco et al. 2011; Tedesco et al. 2012).

The other example is Globoid cell leukodystrophy (also known as Krabbe’s disease), which is an autosomal recessively inherited disease caused by a deficiency of galactocerebrosidase (GALC), a lysosomal enzyme that degrades galactosylceramide, a major glycolipid component of myelin and myelin-forming cells (Katona et al. 2008). In an experimental model for the treatment of Krabbe’s disease, Katona and colleagues showed that the life span was increased in chimeric model mice when wild-type ES cells with a SATAC containing the human *GALC* gene was microinjected into the model mouse-derived blastocysts. However, the life-span extension is possibly attributable to the genomic copy of the *GALC* gene present in the wild-type ES cells.

Finally, advances in the efficiency of methods used for the differentiation and purification of stem cells, including ES and iPS cells, are anticipated, and the application of these methods to ES/iPS cells combined with HAC vector systems may enable the development of more sophisticated gene therapies. Thus, stem cells, potentially derived from multiple sources, combined with HAC-mediated gene delivery, should permit safe treatment of various genetic defects. The next step in the future of gene therapy is to demonstrate functional restoration and safety in vivo using large animal models such as dogs and monkeys.

## Conclusions

The chromosomal vector systems offer complementary and desirable characteristics for use as gene delivery vectors to overcome various problems in existing viral and non-viral vector systems. The most important property of HAC and MAC vectors is that they can express entire complex signaling pathways under their normal physiological regulation, which is of great potential benefit. Stem cells possess two characteristic features: the ability for self-renewal and the ability for multi-lineage differentiation. A number of other applications of HACs/MACs in addition to gene therapy and animal models are possible (Fig. 3). For example, HACs can be used for basic research on human cells and gene therapy, and MACs for basic research on mouse cells and Tc mice. There is also increasing interest in HACs/MACs as a potential platform for developing more sophisticated control of mammalian cells in the new area of “Synthetic Biology,” e.g., studying the RNA world. Thus, HACs/MACs may be designated as “multipotent vectors.”

A combination of chromosome engineering technologies and genome-editing technologies should facilitate the applications to bio-medical challenges (Fig. 3). Genome editing using ZFNs, transcription activator-like effector nucleases (TALENs), or CRISPR/Cas9 are efficient strategies for the modification of desired endogenous genes in cells and organisms (Hsu et al. 2014; Sander and Joung 2014). In principle, a combination of HAC/MAC/chromosome transfer and genome-editing technologies has several advantages for the generation of humanized animal models and disease models as well as for basic MMCT and chromosome engineering technologies. Possible application of the combined technologies was discussed in the text. Genome-editing technologies have been applied to many organisms due to the simple mRNA and plasmid transfection techniques. To our knowledge, HAC/MAC have never been transferred to plants and insects. If HAC/MAC can be transferred to several organisms in addition to mammalian cells, HAC/MAC may be widely utilized in many research areas such as genome editing. Recently, we developed fusion chromosomes of human-plants via whole cell fusion technique (unpublished data). These chromosomes may be used as shuttle vectors or multipurpose vectors, if they segregate in plant and mammalian cells. Shuttle chromosome vectors that can be transferred to any organisms and segregate in them need to be developed.

## Abbreviations

6-TG: 6-thioguanine
BAC: Bacterial artificial chromosome
BRCA-1: Breast cancer susceptibility gene I
BRMS1: Breast cancer metastasis-suppressor 1
CENP-A: Centromere protein A
CHO: Chinese hamster ovary
CRISPR: Clustered regulatory interspaced short palindromic repeat
CSN2: Beta-casein
CYP3A: Cytochrome P450, family 3, subfamily A
CYP3A-HAC: CYP3A cluster-containing HAC
DMD: Duchenne musclular dystrophy
DS: Down syndrome
DYS-HAC: Genomic dystrophin locus-containing HAC
EGFP: Enhanced green fluorescent protein
ES: Embryonic stem
FVIII: Human factor VIII
GALC: Galactosylceramidase
GATA1s: GATA-binding protein 1 shorter isoform
GCH1: GTP cyclohydrolase 1
H/F protein: Hemagglutinin/fusion protein
HAC: Human artificial chromosome
HAT: Hypoxanthine-aminopterin-thymidine
hCFs: Human chromosome fragments
hiMSC: Human immortalized mesenchymal stem cell
HLA: Human Leukocyte Antigen
HPRT: Hypoxanthine phosphoribosyl transferase
iHAC: iPS cell-inducible HAC
iPS: Induced pluripotent stem
LIT1(KCNQ1OT1): Imprinted antisense RNA in the human KvLQT1
MAC: Mouse artificial chromosome
MEFs: Mouse embryonic fibroblasts
mGS cells: Multipotent germline stem cells
MI-HAC: Multi-integrase HAC
MMCT: Microcell-mediated chromosome transfer
MUC4: Mucin 4
MV: Measles virus
NBS1: Nijmegen breakage syndrome 1
OPN: Osteopontin
PAC: P1-derived artificial chromosome
PDLs: Population doubling levels
PEG: Polyethylene glycol
PITX1: Paired-like homeodomain 1
SATAC: Satellite-DNA-based artificial chromosome
scFv: Single-chain variable fragment
SIM: Simultaneous or sequential integration of multiple gene-loading vectors
ssODN: Single-strand donor oligonucleotides
TALE: Transactivator like effector
TALENs: Transactivator like effector nucleases
TAM: Transient abnormal myelopoiesis
TAR: Transformation-associated recombination
Tc mouse: Trans-chromosomic mouse
tet-O: Tetracycline operator
TfR: Transferrin receptor
Ts21: Trisomy 21
VHL: Von Hippel–Lindau tumor suppressor
YAC: Yeast artificial chromosome
ZFP: Zinc finger protein
ZFNs: Zinc finger nucleases
